# Redescription and revision of the Neotropical genus *Pseudoheptascelio* Szabó (Hymenoptera, Platygastridae, Scelioninae), parasitoids of eggs of short-horned grasshoppers (Orthoptera, Acrididae)

**DOI:** 10.3897/zookeys.136.1580

**Published:** 2011-10-12

**Authors:** Norman F. Johnson, Luciana Musetti

**Affiliations:** 1 Department of Evolution, Ecology and Organismal Biology, The Ohio State University, 1315 Kinnear Road, Columbus, Ohio 43212, U.S.A.

**Keywords:** Platygastridae, Platygastroidea, Scelioninae, egg-parasitoid, *Cornops*, key, biological control, water hyacinth, *Eichhornia*

## Abstract

The genus *Pseudoheptascelio* Szabó is redescribed and its species revised. We recognize four species: *Pseudoheptascelio muesebecki* Szabó, *Pseudoheptascelio cornopis* Masner, *Pseudoheptascelio tico* **sp. n.** and *Pseudoheptascelio rex* **sp. n.** The genus is found from Guatemala south to the Brazilian state of Rio Grande do Sul. The species *Pseudoheptascelio cornopis* is recorded as a parasitoid of the eggs of *Cornops aquaticum* (Bruner) on water hyacinth, *Eichhornia crassipes* (Mart.) Solms.

## Introduction

The genus *Pseudoheptascelio* was described by Szabó (1966) from a single female collected in the state of Pará in northern Brazil. Masner (1972) subsequently erected *Tanaoscelio* for a single species collected in Trinidad and recorded as attacking the eggs of *Cornops longicorne* (Bruner) (Orthoptera: Acrididae, Leptysminae), a grasshopper that was being studied as a potential biological control agent for water hyacinth, *Eichhornia crassipes* (Mart.) Solms (Commelinales: Pontederiaceae). Masner (1976) later discovered an error in Szabó’s original description concerning the presence of a complete radial vein in the hind wing. In fact, the tubular portion of the vein is abbreviated and does not reach the costal margin of the wing. Therefore, Masner concluded that these two taxonomic concepts were equivalent.

*Pseudoheptascelio* is found only in the New World tropics, from Belize and Guatemala south to southeastern Brazil. The distribution of the only known host, *Cornops*, is very similar, although its range extends north along the coasts of Mexico (Adis et al. 2007). Developments in our understanding of this group of grasshoppers subsequent to the original description of *Tanaoscelio* (Roberts & Carbonell, 1979) suggest that the species identification of the host should be updated. *Cornops longicorne* is now considered to be a junior synonym of *Cornops frenatum* (Marschall). This latter species, however, is terrestrial and its host plants are unknown (Roberts and Carbonell 1979). The only semi-aquatic species attacking *Eichhornia* in Trinidad appears to be *Cornops aquaticum* (Bruner) (Roberts and Carbonell 1979, Adis et al. 2007).

## Materials and methods

This work is based upon specimens in the following collections, with abbreviations used in the text: AEIC, American Entomological Institute, Gainesville, FL^1^; BMNH, The Natural History Museum, London, UK^2^; BPBM, Bernice P. Bishop Museum, Honolulu, HI^3^; CNCI, Canadian National Collection of Insects, Ottawa, Canada^4^; HNHM, Hungarian Natural History Museum, Budapest, Hungary^5^; MIZA, Museo del Instituto de Zoología Agrícola, Maracay, Venezuela^6^; OSUC, C.A. Triplehorn Insect Collection, Ohio State University, Columbus, OH^7^; TAMU, Texas A&M University Insect Collection, College Station, TX^8^; USNM, National Museum of Natural History, Washington, DC^9^.

Abbreviations and morphological terms used in text: A1, A2, ... A12: antennomere 1, 2, … 12; claval formula: distribution of the large, multiporous basiconic sensilla on the underside of apical antennomeres of the female, with the segment interval specified followed by the number of sensilla per segment (Bin 1981); EH: eye height, length of compound eye measured parallel to dorsoventral midline of head; IOS: interocular space, minimal distance on frons between compound eyes; OD: ocellar diameter, greatest width of ocellus; OOL: ocular ocellar line, the shortest distance from inner orbit and outer margin of lateral ocellus (Masner 1980); T1, T2, ... T7: metasomal tergite 1, 2, ... 7; S1, S2, … S7: metasomal sternite 1, 2, … 7. Morphological terminology otherwise follows Masner (1980) and Mikó et al. (2007).

Appendix 1 lists terms associated with identifiers in the Hymenoptera Anatomy Ontology (Yoder et al. 2010). Identifiers in the format HAO_XXXXXXX represent concepts in the HAO version 2011-07-14 and are provided to enable readers to confirm their understanding of the concepts being referenced. To find out more about a given concept use the identifier as a search term at http://glossary.hymao.org. The identifier can also be used as a URI (universal resource identifier) by appending the identifier to 'http://purl.obolibrary.org/obo/' (e.g. http://purl.obolibrary.org/obo/HAO_0000124). URLs in the format http://purl.org/net/hao/HAO_0123456 resolve to the HAO’s community-based resource that includes additional images, notes, and other metadata.

In the Material Examined section the numbers prefixed with “OSUC” are unique identifiers for the individual specimens. The label data for all specimens have been georeferenced and recorded in the Hymenoptera On-Line database, and details on the data associated with these specimens can be accessed at the following link, hol.osu.edu, and entering the identifier in the form. Note the space between the acronym and the number.

Data associated with the genus *Pseudoheptascelio* can be accessed at http://hol.osu.edu/index.html?id=548. The generic and species descriptions were generated using a database application, vSysLab, designed to facilitate the production of a taxon by character data matrix, and to integrate those data with the existing taxonomic and specimen-level database. Data may be exported in both text format and as input files for other applications. The text output for descriptions is in the format of "Character: Character state (s)". Images and measurements were made using AutoMontage and Cartograph extended-focus software, using JVC KY-F75U digital camera, Leica Z16 APOA microscope, and 1X objectve lens. A standard set of images is provided for each species: dorsal habitus, lateral habitus, dorsal and lateral views of the head and mesosoma, and anterior view of head. Images are archived at Morphbank (www.morphbank.net) and in Specimage (specimage.osu.edu), the image database at The Ohio State University.

The electronic version of the paper contains hyperlinks to external resources. Insofar as possible, the external information conforms to standards developed and maintained through the organization Biodiversity Information Standards (Taxonomic Database Working Group). All new species have been prospectively registered with Zoobank (Polaszek et al. 2005, www.zoobank.org), and other taxonomic names, where appropriate, have been retrospectively registered. The external hyperlinks are explicitly cited in the endnotes so that users of the printed version of this article have access to the same resources. Life sciences identifiers, LSIDs, may be resolved at the specified URLs or at lsid.tdwg.org.

This work is conducted as part of the Platygastroidea Planetary Biodiversity Inventory. The authors made equal contributions.

## Taxonomy

### Pseudoheptascelio

Szabó

urn:lsid:zoobank.org:act:50643EC2-1EF4-496D-937B-8FBFDC9F9B8E

urn:lsid:biosci.ohio-state.edu:osuc_concepts:548

http://species-id.net/wiki/Pseudoheptascelio

Pseudoheptascelio Szabó, 1966: 166 (original description. Type: *Pseudoheptascelio muesebecki* Szabó, by monotypy and original designation); Masner 1976: 18 (description, key to species); De Santis 1980: 315 (catalog of species of Brazil); Johnson 1992: 467 (catalog of world species); Loiácono and Margaría 2002: 558 (catalog of Brazilian species). urn:lsid:zoobank.org:act:EF9EA824-0219-42C1-A057-055D8E11FE8 urn:lsid:biosci.ohio-state.edu:osuc_concepts:9625
Tanaoscelio Masner, 1972: 1213 (original description. Type: *Tanaoscelio cornopis* Masner, by monotypy and original designation); Masner 1976: 18 (junior synonym of *Pseudoheptascelio* Szabó).

#### Description.

 Body length: 4.09–5.45 mm (n=81).

**Head.** Head shape in dorsal view: weakly transverse, width approximately 1.5× greatest length. Hyperoccipital carina: absent. Occipital carina: present laterally, broadly interrupted medially. Occipital carina sculpture: crenulate. OOL: lateral ocellus nearly contiguous with inner orbits, OOL < 0.5 OD. Upper portion of frons: convex, without frontal shelf. Scrobe shape: frons with shallow unmargined depression above toruli. Frons sculpture: areolate rugose, transversely striate within scrobe. Submedian carina: absent. Orbital carina: absent. Inner orbits: diverging ventrally. IOS/EH: IOS slightly less than EH. Interantennal process: rounded, strongly developed. Central keel: absent. Torulus opening: laterally on interantennal process. Lower frons striae: absent. Malar sulcus: present. Compound eye size: of normal proportions, not significantly reduced. Compound eye setation: sparsely setose. Gena: broad, convex, distinctly produced behind eye. Clypeus shape: transversely rectangular. Apical margin of clypeus: straight. Anteclypeus: present, delimited dorsally by raised carina. Postclypeus: present, strongly transverse. Labrum: not visible, hidden behind clypeus. Mandible shape: short, inconspicuous. Mandibular teeth: apex with 2, acute, subequal teeth. Arrangement of mandibular teeth: transverse. Number of maxillary palpomeres: 4. Shape of maxillary palpomeres: cylindrical. Number of labial palpomeres: 2.

**Antenna.** Number of antennomeres in female: 12. Number of antennomeres in male: 10. Insertion of radicle into A1: parallel to longitudinal axis of A1. Shape of A1: more or less cylindrical, not flattened. Length of A3 of female: distinctly longer than A2. Number of clavomeres in female antenna: 7. Claval formula of female antenna: A12–A7/1-2-2-2-2-2. Arrangement of doubled multiporous plate sensilla on female clava: in longitudinal pairs. Tyloid distribution on male antenna: A5 only. Shape of male flagellum: subclavate.

**Mesosoma.** Mesosoma shape in dorsal view: longer than wide. Mesosoma shape in lateral view: longer than high. Medial portion of transverse pronotal carina: weakly indicated laterally. Posterior apex of pronotum in dorsal view: straight, bifid apically to articulate with tegula. Vertical epomial carina: present. Dorsal epomial carina (corresponding to lateral portion of transverse pronotal carina of Vilhelmsen et al. 2010): present. Anterior face of pronotum: oblique, visible dorsally, short. Lateral face of pronotum: weakly concave below dorsal epomial carina. Netrion: present. Netrion shape: moderately wide, closed ventrally. Anterior portion of mesoscutum: vertical, flexed ventrally to meet pronotum. Mesoscutum shape: semielliptical, excavate at base of wings. Skaphion: absent. Notauli: present, percurrent. Parapsidal lines: absent. Admedial lines: absent. Transscutal articulation: well-developed, wide, bridged by 6–10 trabecula. Shape of mesoscutellum: quadrate to trapezoidal. Armature of mesoscutellum: axillula produced posteriorly into short, broad spines. Surface of mesoscutellum: convex anteriorly, depressed posteriorly. Median longitudinal furrow on mesoscutellum: absent. Shape of axillula: large, triangular, extending length of mesoscutellum. Metascutellum: clearly differentiated. Metascutellar armature: produced medially into short, shallowly bidentate process. Metapostnotum: not delimited externally. Extent of metasomal depression of propodeum: percurrent, extending anteriorly to anterior margin of propodeum. Lateral propodeal projection: well-developed, extending clearly beyond anterior margin of T1. Mesopleural carina: absent or strongly abbreviated, present only near mid coxa. Mesal portion of acetabular carina: projecting anteriorly, not separating fore coxae. Mesopleural pit: present. Sternaulus: absent. Posterodorsal corner of mesopleuron: rounded anteriorly.

**Legs.** Number of mid tibial spurs: 1. Number of hind tibial spurs: 1. Dorsal surface of hind coxa: smooth. Hind tibia shape: cylindrical, ecarinate. Trochantellus: indicated only as basal swelling of femur.

**Wings.** Wing development of female: macropterous. Wing development of male: macropterous. Tubular veins in fore wing: present. Bulla of fore wing R: absent. Extent of marginal venation of fore wing: R1 reaching and ending at costal margin. Origin of r-rs in fore wing: arising before (basad of) R/R1 attains costal margin. Development of basal vein (Rs+M) in fore wing: spectral. Development of R in hind wing: abbreviated, not attaining costal margin.

**Metasoma.** Number of externally visible terga in female: 6. Number of externally visible sterna in female: 6. Number of externally visible terga in male: 7. Number of externally visible sterna in male: 7. Shape of metasoma: lanceolate. Laterotergites: present, narrow. Laterosternites: present. T1 of female: raised medially into low, rectangular platform, laterally depressed. Relative size of metasomal tergites: T2–T4 largest, subequal in size. Terga with basal crenulae: T1–T3. Sublateral carinae on tergites: present on T1–T4. Median longitudinal carina on metasomal tergites: present T2–T3, variably extending beyond. Anterior margin of S1: protruding anteriorly as short sharp extension of median longitudinal carina of S1. Distribution of felt fields: present on S2, S3. Ovipositor type: Scelio-type (Austin and Field 1997).

#### Diagnosis.

**Figures 1–6.**
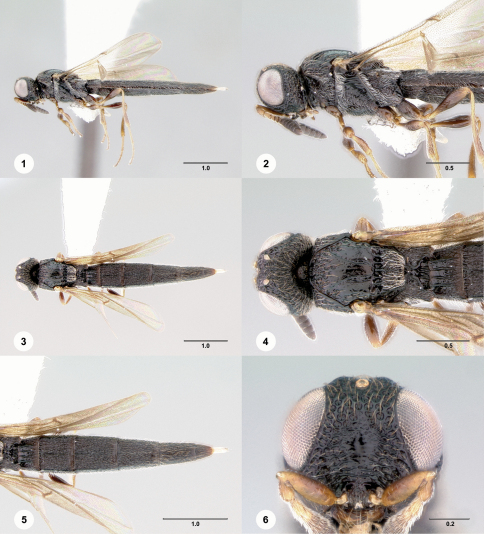
**^30^** *Pseudoheptascelio cornopis* (Masner), female (OSUC 186250) **1** Lateral habitus **2** Head and mesosoma, lateral view **3** Dorsal habitus **4** Head and mesosoma, dorsal view **5** Metasoma, dorsal view **6** Head, anterior view. Scale bars in millimeters.

 Within the tribe Scelionini *s. str.* the genera *Pseudoheptascelio*, *Scelio*, *Sceliocerdo*, and *Synoditella* have 10-segmented antennae in the male. *Pseudoheptascelio* may be separated from the vast majority of these species by the presence of short, hooklike axillular projections on the mesoscutellum, the medially produced metascutellum, the densely setose anterior margins of both the mesopleuron and metapleuron (Figs 2, 12, 18, 24), the rigid unflexed metasoma (Figs 1, 11, 17, 23), well-developed notauli (Figs 4, 14, 20, 26), the absence of fanlike striae arising from the base of the mandible (Figs 6, 15, 21, 27), and the broadly interrupted occipital carina (Figs 4, 14, 20, 26). At least one Neotropical species of *Scelio* has axillular points and a projecting metascutellum. *Pseudoheptascelio* may be distinguished from this by the posteriorly declivous mesoscutellum, distinct notauli, the presence of dense pilosity on the anterior margins of the meso- and metapleuron, the subclavate male antenna (Fig. 9), the elongate T2–T6 (clearly longer than wide), and the smooth transition of the lateral margins of T5–T7 and subclavate antenna in the male (Fig. 8).

#### Key to species

**Table:** 

1	T2–T3 reticulate (Figs 5, 13, 19), without distinct longitudinal rugulae; mesosoma black; T6 longer than wide basally	2
–	T2–T3 with distinct longitudinal rugulae (Fig. 25); mesosoma often with reddish portions (Figs 23–26); basal width of T6 greater than its length	3
2	Occiput without microsculpture within foveolae, appearing shining; length of T5 1.3–1.8 x its maximum width (Fig. 16)	*Pseudoheptascelio muesebecki*
–	Occiput with dense fine microsculpture within foveolae, appearing matte (Fig. 10); length of T5 1.6–2.2 x its maximum width (Fig. 7)	*Pseudoheptascelio cornopis*
3	Head and mesosoma without coriaceous microsculpture, appearing shining; metascutellum short, subquadrate (Fig. 20)	*Pseudoheptascelio rex*
–	Head and mesosoma with distinct superimposed coriaceous microsculpture, giving body overall matte appearance; metascutellum distinctly longer than wide (Fig. 26)	*Pseudoheptascelio tico*

### Pseudoheptascelio cornopis

(Masner)

urn:lsid:zoobank.org:act:270062C9-88EC-4138-8ADF-284DF6B24F93

urn:lsid:biosci.ohio-state.edu:osuc_concepts:5132

http://species-id.net/wiki/Pseudoheptascelio_cornopis

Figures 1 10 Morphbank^10^

Tanaoscelio cornopis Masner, 1972: 1214 (original description).
Pseudoheptascelio cornopis (Masner): Masner, 1976: 18 (generic transfer).

#### Description.

 Body length of female: 4.37–5.45 mm (n=11). Body length of male: 4.58–5.22 mm (n=4). Mesosoma color: black. Body microsculpture pattern: smooth.

Rugae on occiput: reticulate. Microsculpture between occipital rugae: foveolate (Fig. 10). Setae on crests of occipital rugae: absent. Shape of female A4: length subequal to width. Shape of female A5: transverse. Shape of female A6: distinctly transverse.

Setation of pronotal depression: moderately to densely setose. Setation of netrion: moderately to densely setose (Fig. 2). Sculpture of midlobe of mesoscutum: foveate to areolate anteriorly, sculpture effaced, sparser posteriorly (Figs 4, 10). Number of trabecula across transscutal articulation: 7–8, widely spaced. Shape of metascutellum: short, shallowly cleft medially (Fig. 4). Sculpture of mesopleural depression: almost entirely sculptured, with transverse rugulae and interspersed irregular fovea.

Sculpture of T2–T3: irregularly reticulate, without longitudinal orientation. Length/width of female T5: 1.61–2.22 mm (n=12). Length/width of female T6: 1.10–1.50 mm (n=11). Sculpture of T6: with reticulate microsculpture only. Apex of male T7: pointed laterally, shallowly excavate or straight medially (Fig. 8).

**Figures 7–10.**
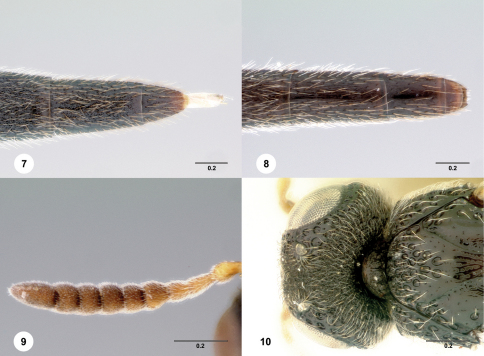
**^31^** *Pseudoheptascelio cornopis* (Masner) **1** Apex of metasoma, female, dorsal view (OSUC 186250) **2** Apex of metasoma, male (OSUC 248318) **3** Antenna, segments 2–10, male (OSUC 248318) **4**, Head, dorsal view, holotype female (B.M. TYPE HYM. 9.772). Scale bars in millimeters.

#### Diagnosis.

*Pseudoheptascelio cornopis* is distinguished from *Pseudoheptascelio muesebecki* by the densely and finely sculptured vertex and the more elongate T5 (length/width 1.6–2.2).

#### Link to Distribution Map.

^11^ [http://hol.osu.edu/map-full.html?id=5132]

#### Associations.

 Data from specimen labels: emerged from egg of *Cornops* Scudder: [Orthoptera: Acrididae]; solitary egg parasitoid of *Cornops* Scudder: [Orthoptera: Acrididae]; unspecified association *Cornops frenatum* (Marschall): [Orthoptera: Acrididae]; emerged from egg of *Cornops longicorne* (Brunner): [Orthoptera: Acrididae]; solitary egg parasitoid of *Cornops longicorne* (Brunner): [Orthoptera: Acrididae]; emerged from egg on *Eichhornia crassipes* (Mart.): [Liliales: Pontederiaceae]; solitary egg parasitoid ex *Eichhornia crassipes* (Mart.): [Liliales: Pontederiaceae]; unspecified association *Eichhornia crassipes* (Mart.): [Liliales: Pontederiaceae]

#### Material Examined.

*Holotype*, female, *Tanaoscelio cornopis*: **TRINIDAD AND TOBAGO**: Trinidad Isl., Débé, V-1970, B.M. TYPE HYM. 9.772 (deposited in BMNH). *Paratypes*: **TRINIDAD AND TOBAGO:** 3 females, 1 male, 2 unknowns, BMNH(E)#790244–790245 (BMNH); OSUC 186160–186162 (CNCI); OSUC 248318 (USNM). *Other material*: (9 females, 2 males) **BOLIVIA:** 7 females, 1 male, OSUC 186242, 186245–186250, 186253 (CNCI). **BRAZIL:** 1 female, 1 male, OSUC 186241 (CNCI); OSUC 131887 (OSUC). **GUYANA:** 1 female, OSUC 215796 (BPBM). Allotype: **TRINIDAD AND TOBAGO:** 1 male, BMNH(E)#790243 (BMNH). **VENEZUELA:** 1 female, OSUC 221615 (MIZA).

#### Comments.

 In the brief key to species Masner (1976) stated that the stigmal vein (r-rs) is embedded in a milky spot, forming a pseudostigma. The species *Pseudoheptascelio muesebecki*, in contrast, was characterized as having the area around the stigmal vein transparent. We find that there is considerable variability in the development of the pseudostigma and that it is present in all specimens of *Pseudoheptascelio*.

### Pseudoheptascelio muesebecki

Szabó

urn:lsid:zoobank.org:act:E3EF612E-195C-45DD-86BA-14EB72125754

urn:lsid:biosci.ohio-state.edu:osuc_concepts:5133

http://species-id.net/wiki/Pseudoheptascelio_muesebecki

**Figures 11–16.**
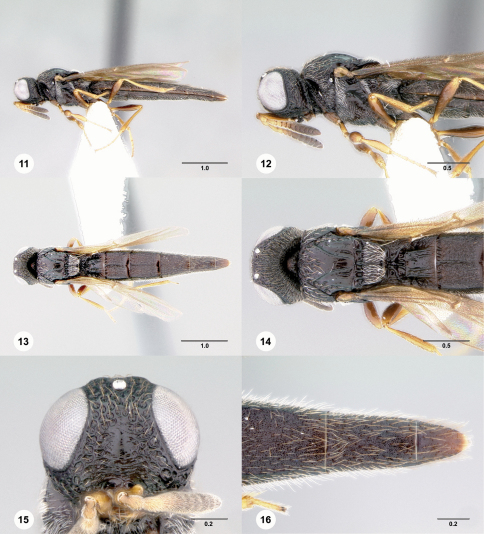
**^32^** *Pseudoheptascelio muesebecki* Szabó, female (OSUC 186208) **11** Lateral habitus **12** Head and mesosoma, lateral view **13** Dorsal habitus **14** Head and mesosoma, dorsal view **15** Head, anterior view **16** Apex of metasoma, dorsal view. Scale bars in millimeters.

Figures 11–16 Morphbank^12^

Pseudoheptascelio muesebecki Szabó, 1966: 167 (original description); Masner, 1976: 18 (type information).

#### Description.

 Body length of female: 4.09–5.42 mm (n=15). Mesosoma color: black. Body microsculpture pattern: smooth.

Rugae on occiput: reticulate. Microsculpture between occipital rugae: absent. Setae on crests of occipital rugae: absent. Shape of female A4: length subequal to width. Shape of female A5: transverse. Shape of female A6: distinctly transverse.

Setation of pronotal depression: moderately to densely setose (Fig. 12). Setation of netrion: moderately to densely setose. Sculpture of midlobe of mesoscutum: foveate to areolate throughout; foveate to areolate anteriorly, or sculpture effaced, sparser posteriorly (Fig. 14). Number of trabecula across transscutal articulation: 7–8, widely spaced. Shape of metascutellum: short, shallowly cleft medially (Fig. 14). Sculpture of mesopleural depression: almost entirely sculptured, with transverse rugulae and interspersed irregular fovea.

Sculpture of T2–T3: irregularly reticulate, without longitudinal orientation. Length/width of female T5: 1.26–1.80 mm (n=15). Length/width of female T6: 1.05–1.53 mm (n=15). Sculpture of T6: with reticulate microsculpture only.

#### Diagnosis.

 This species is very similar to *Pseudoheptascelio cornopis*, and it may be distinguished by the less elongate T5 and the coarse areolate sculpture on the vertex.

#### Link to Distribution Map.

^13^ [http://hol.osu.edu/map-full.html?id=5133]

#### Assocations.

No data available.

#### Material Examined.

*Holotype*, female: **BRAZIL**: PA, Belém, no date, E. Horváth, HNHM 0015 (deposited in HNHM). *Other material*: (14 females) **BOLIVIA**: 1 female, OSUC 186244 (CNCI). **BRAZIL**: 9 females, OSUC 186233–186240 (CNCI); OSUC 58878 (OSUC). **ECUADOR**: 1 female, OSUC 186208 (CNCI). **PARAGUAY**: 2 females, OSUC 176024, 176033 (OSUC). **TRINIDAD AND TOBAGO**: 1 female, OSUC 186163 (CNCI). 

### Pseudoheptascelio rex

Johnson & Musetti sp. n.

urn:lsid:zoobank.org:act:32540FD3-5763-4536-BD1A-A50849D8A6D6

urn:lsid:biosci.ohio-state.edu:osuc_concepts:242983

http://species-id.net/wiki/Pseudoheptascelio_rex

**Figures 17–22.**
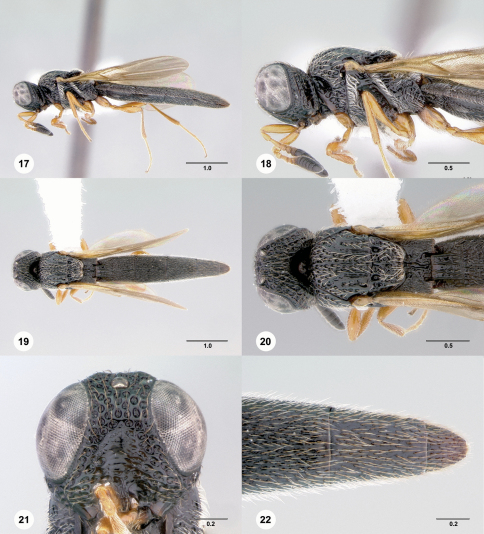
**^33^** *Pseudoheptascelio rex*, sp. n., holotype female (OSUC 186230) **17** Lateral habitus **18** Head and mesosoma, lateral view **19** Dorsal habitus **20** Head and mesosoma, dorsal view **21** Head, anterior view **22** Apex of metasoma, dorsal view. Scale bars in millimeters.

Figures 17–22 Morphbank^14^

#### Description.

 Body length of female: 4.13–5.26 mm (n=20). Body length of male: 4.50–5.16 mm (n=13). Mesosoma color: black; red brown at least dorsally, otherwise dark to brown black. Body microsculpture pattern: smooth.

Rugae on occiput: longitudinal. Microsculpture between occipital rugae: absent. Setae on crests of occipital rugae: present. Shape of female A4: length subequal to width; length distinctly greater than width. Shape of female A5: transverse; subquadrate. Shape of female A6: distinctly transverse; weakly transverse.

Setation of pronotal depression: glabrous or sparsely setose. Setation of netrion: moderately to densely setose. Sculpture of midlobe of mesoscutum: foveate to areolate throughout (Fig. 20). Number of trabecula across transscutal articulation: 7–8, widely spaced. Shape of metascutellum: short, shallowly cleft medially (Fig. 20). Sculpture of mesopleural depression: foveolate anteriorly, transversely rugulose ventrally, with large smooth area dorsally surrounding mesopleural pit.

Sculpture of T2–T3: reticulate, with distinct longitudinal orientation. Length/width of female T5: 0.89–1.72 mm (n=20). Length/width of female T6: 0.81–1.26 mm (n=20). Sculpture of T6: with shallow foveolae impressed on reticulate background microsculpture. Apex of male T7: pointed laterally, shallowly excavate or straight medially.

#### Diagnosis.

 This species shares the short female T6 (Fig. 22) and, in many specimens, the red mesosoma with *Pseudoheptascelio tico*. It may be distinguished by the short metascutellum (Fig. 20) and the absence of coriaceous microsculpture on the head and mesosoma.

#### Etymology.

 The specific epithet is Latin for king and should be treated as a noun in apposition.

#### Link to Distribution Map.

^15^ [http://hol.osu.edu/map-full.html?id=242983]

#### Associations.

 Data from specimen labels: collected on *Trichocentrum panamensis* Rolfe: [Orchidales: Orchidaceae]

#### Material Examined.

*Holotype*, female: **ECUADOR:** Sucumbíos Prov., Sacha Lodge, 00°30'S, 76°30'W, 270m, 27.VIII–10.IX.1995, malaise trap, P. Hibbs, OSUC 186230 (deposited in CNCI). *Paratypes*: (49 females, 14 males) **BOLIVIA:** 6 females, 1 male, OSUC 186251–186252, 186254–186258 (CNCI). **COLOMBIA:** 10 females, 2 males, OSUC 287928 (CNCI); OSUC 210338–210341 (FSCA); OSUC 144252–144253, 189092, 191363, 193964, 210336, 224326 (OSUC). **COSTA RICA:** 2 females, OSUC 186186, 186194 (CNCI). **ECUADOR:** 19 females, 9 males, OSUC 186203–186207, 186209–186229, 186231 (CNCI); OSUC 58879 (OSUC). **FRENCH GUIANA:** 2 females, OSUC 186202, 287926 (CNCI). **GUYANA:** 1 male, OSUC 215795 (BPBM). **NICARAGUA:** 1 male, OSUC 320737 (TAMU). **PANAMA:** 9 females, OSUC 186199–186200 (CNCI); OSUC 248311–248317 (USNM). **PERU:** 1 female, OSUC 186232 (CNCI).

### Pseudoheptascelio tico

Johnson & Musetti sp. n.

urn:lsid:zoobank.org:act:23DBB9A2-4F23-4F7E-AFC8-38A2906EE95E

urn:lsid:biosci.ohio-state.edu:osuc_concepts:242982

http://species-id.net/wiki/Pseudoheptascelio_tico

**Figures 23–28.**
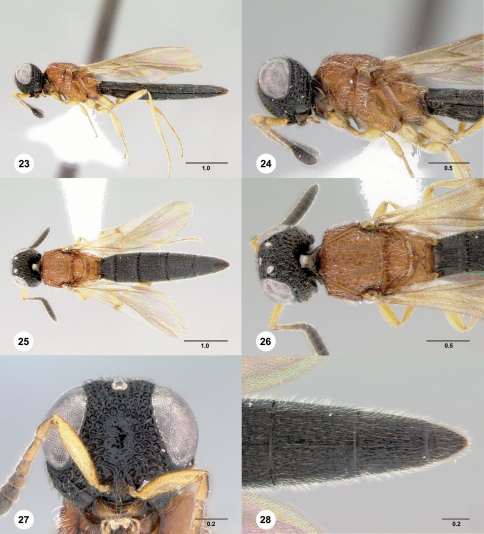
**^34^** *Pseudoheptascelio tico*, n.sp., holotype female (OSUC 186191) **23** Lateral habitus **24** Head and mesosoma, lateral view **25** Dorsal habitus **26** Head and mesosoma, dorsal view **27** Head, anterior view **28** Apex of metasoma, dorsal view. Scale bars in millimeters.

Figures 23–28 Morphbank^16^

#### Description.

 Body length of female: 4.44–5.14 mm (n=12). Body length of male: 4.42–4.85 mm (n=5). Mesosoma color: red brown at least dorsally, otherwise dark to brown black. Body microsculpture pattern: with widespread superimposed coriaceous microsculpture.

Rugae on occiput: reticulate. Microsculpture between occipital rugae: absent. Setae on crests of occipital rugae: absent. Shape of female A4: length distinctly greater than width. Shape of female A5: subquadrate. Shape of female A6: weakly transverse.

Setation of pronotal depression: glabrous or sparsely setose (Fig. 24). Setation of netrion: glabrous or sparsely setose. Sculpture of midlobe of mesoscutum: foveate to areolate throughout (Fig. 26). Number of trabecula across transscutal articulation: 9–11, closely spaced. Shape of metascutellum: distinctly elongate, deeply cleft medially. Sculpture of mesopleural depression: irregularly foveolate, transverse rugulae very weakly indicated.

Sculpture of T2–T3: reticulate, with distinct longitudinal orientation. Length/width of female T5: 0.97–1.16 mm (n=13). Length/width of female T6: 0.93–1.13 mm (n=13). Sculpture of T6: with shallow foveolae impressed on reticulate background microsculpture. Apex of male T7: weakly pointed laterally, distinctly sinuous medially.

#### Diagnosis.

 This species should only be confused with red specimens of *Pseudoheptascelio rex*. It may be distinguished by the well-developed coriaceous microsculpture on the head and mesosoma, and the elongate, deeply cleft metascutellum (Fig. 26).

#### Etymology.

 The specific epithet is a colloquial term for a Costa Rican, reflecting the origin of most of the specimens we have seen. It should be treated as a noun in apposition.

#### Link to Distribution Map.

^17^ [http://hol.osu.edu/map-full.html?id=242982]

#### Assocations.

No data available.

#### Material Examined.

*Holotype*, female: **COSTA RICA:** Alajuela Prov., creekbed, San Ramón Biological Station, 700m, 24.III–26.III.1996, yellow pan trap, L. Masner, OSUC 186191 (deposited in CNCI). *Paratypes*: (12 females, 5 males) **BELIZE:** 1 female, OSUC 287927 (USNM). **COSTA RICA:** 9 females, 5 males, OSUC 186181–186185, 186187–186190, 186192, 186195–186198 (CNCI). **GUATEMALA:** 1 female, OSUC 186268 (AEIC). PANAMA: 1 female, OSUC 186201 (CNCI).

## Supplementary Material

XML Treatment for Pseudoheptascelio

XML Treatment for Pseudoheptascelio cornopis

XML Treatment for Pseudoheptascelio muesebecki

XML Treatment for Pseudoheptascelio rex

XML Treatment for Pseudoheptascelio tico

## Endnotes

1 http://biocol.org/urn:lsid:biocol.org:col:1008

2 http://biocol.org/urn:lsid:biocol.org:col:1009

3 http://biocol.org/urn:lsid:biocol.org:col:1010

4 http://biocol.org/urn:lsid:biocol.org:col:1012

5 http://biocol.org/urn:lsid:biocol.org:col:33453

6 http://biocol.org/urn:lsid:biocol.org:col:33834

7 http://biocol.org/urn:lsid:biocol.org:col:1014

8 http://biocol.org/urn:lsid:biocol.org:col:34336

9 http://biocol.org/urn:lsid:biocol.org:col:1019

10 http://www.morphbank.net/?id=644094

11 http://hol.osu.edu/map-full.html?id=5132

12 http://www.morphbank.net/?id=644091

13 http://hol.osu.edu/map-full.html?id=5133

14 http://www.morphbank.net/?id=644092

15 http://hol.osu.edu/map-full.html?id=242983

16 http://www.morphbank.net/?id=644093

17 http://hol.osu.edu/map-full.html?id=242982

18 http://lsid.tdwg.org/urn:lsid:biosci.ohio-state.edu:osuc_pubs:20940

19 http://lsid.tdwg.org/urn:lsid:biosci.ohio-state.edu:osuc_pubs:131

20 http://lsid.tdwg.org/urn:lsid:biosci.ohio-state.edu:osuc_pubs:235

21 http://lsid.tdwg.org/urn:lsid:biosci.ohio-state.edu:osuc_pubs:229

22 http://lsid.tdwg.org/urn:lsid:biosci.ohio-state.edu:osuc_pubs:21243

23 http://lsid.tdwg.org/urn:lsid:biosci.ohio-state.edu:osuc_pubs:998

24 http://lsid.tdwg.org/urn:lsid:biosci.ohio-state.edu:osuc_pubs:311

25 http://lsid.tdwg.org/urn:lsid:biosci.ohio-state.edu:osuc_pubs:474

26 http://lsid.tdwg.org/urn:lsid:biosci.ohio-state.edu:osuc_pubs:21300

27 http://lsid.tdwg.org/urn:lsid:biosci.ohio-state.edu:osuc_pubs:23221

28 http://lsid.tdwg.org/urn:lsid:biosci.ohio-state.edu:osuc_pubs:452

29 doi:10.1371/journal.pone.0015991

30 http://www.morphbank.net/?id=644067

31 http://www.morphbank.net/?id=644072

32 http://www.morphbank.net/?id=644091

33 http://www.morphbank.net/?id=644092

34 http://www.morphbank.net/?id=644093

## Appendix 1

Correspondence between anatomical terms used and the Hymenoptera Anatomy Ontology. Identifiers may be resolved by appending them to the following URL: http://purl.obolibrary.org/obo/

**Table:** Correspondence between anatomical terms used and the Hymenoptera Anatomy Ontology. Identifiers may be resolved by appending them to the following URL: http://purl.obolibrary.org/obo/

A1	HAO_0000908
A2	HAO_0000706
A3	HAO_0001148
A7	HAO_0001885
A12	HAO_0001884
acetabular carina	HAO_0000292
admedian line	HAO_0000128
anteclypeus	HAO_0000209
antenna	HAO_0000101
antennomere	HAO_0000107
anterior margin of clypeus	HAO_0001767
area	HAO_0000146
articulation	HAO_0001485
axillula	HAO_0000160
basal vein	HAO_0000170
body	HAO_0000182
bulla	HAO_0000184
carina	HAO_0000188
central keel	HAO_0000109
clava	HAO_0001185
clavomere	HAO_0001186
clypeus	HAO_0000212
compound eye	HAO_0000217
corner	HAO_0000223
coxa	HAO_0000228
depression	HAO_0000241
egg	HAO_0000286
epomial carina	HAO_0000307
eye	HAO_0000217
felt field	HAO_0000322
femur	HAO_0000327
flagellum	HAO_0000343
fore wing	HAO_0000351
fovea	HAO_0000241
frons	HAO_0001523
frontal shelf	HAO_0001886
gena	HAO_0000371
head	HAO_0000397
hind coxa	HAO_0000587
hind tibia	HAO_0000631
hind tibial spur	HAO_0001121
hind wing	HAO_0000400
hyperoccipital carina	HAO_0000406
inner orbit	HAO_0000419
interantennal process	HAO_0000422
labrum	HAO_0000456
lateral face of pronotum	HAO_0000483
lateral ocellus	HAO_0000481
laterotergite	HAO_0000493
laterosternite	HAO_0001838
line	HAO_0001586
lower frons striae	HAO_0001770
malar sulcus	HAO_0000504
mandible	HAO_0000506
mandibular tooth	HAO_0001019
margin	HAO_0000510
median longitudinal carina of S1	HAO_0001878
median longitudinal carina on metasomal tergite	HAO_0001878
median longitudinal carina of mesoscutellum	HAO_0001878
mesopleural carina	HAO_0000559
mesopleural pit	HAO_00001358
mesopleuron	HAO_0000566
mesoscutellum	HAO_0000574
mesoscutum	HAO_0001490
mesosoma	HAO_0000576
metapleuron	HAO_0000621
metapostnotum	HAO_0000622
metascutellum	HAO_0000625
metasoma	HAO_0000626
metasomal depression of propodeum	HAO_0000627
mid coxa	HAO_0000635
mid tibial spur	HAO_0001120
midlobe of mesoscutum	HAO_0000520
multiporous plate sensillum	HAO_0000640
netrion	HAO_0000644
notauli (notaulus)	HAO_0000647
occipital carina	HAO_0000653
occiput	HAO_0000658
ocellus	HAO_0000661
ocular ocellar line	HAO_0000662
orbit	HAO_0000672
orbital carina	HAO_0000810
ovipositor	HAO_0000679
palpomere	HAO_0001866
pit	HAO_0000718
parapsidal line	HAO_0000694
postclypeus	HAO_0000743
propodeal lateral projection	HAO_0000763
process	HAO_0000822
projection	HAO_0000829
pronotum	HAO_0000853
propodeum	HAO_0001248
radial vein	HAO_0000888
radicle	HAO_0000889
scrobe	HAO_0000911
sculpture	HAO_0000913
segment	HAO_0000929
skaphion	HAO_0000940
sternaulus	HAO_0001205
sternite	HAO_0001654
submedian carina	HAO_0000973
sulcus	HAO_0000978
tegula	HAO_0000993
tergite	HAO_0001783
tibia	HAO_0001017
torulus	HAO_0001022
transscutal articulation	HAO_0001623
transverse pronotal carina	HAO_0001031
trochantellus	HAO_0001033
tyloid	HAO_0001199
vein	HAO_0001095
venation	HAO_0001096
vertex	HAO_0001077
vertical epomial carina	HAO_0000307
wing	HAO_0001089
